# Effects of probiotics (Vivomixx®) in obese pregnant women and their newborn: study protocol for a randomized controlled trial

**DOI:** 10.1186/s13063-016-1617-5

**Published:** 2016-10-11

**Authors:** Sofie Ingdam Halkjaer, Lisbeth Nilas, Emma Malchau Carlsen, Dina Cortes, Thórhallur Ingi Halldórsson, Sjúrdur Frodi Olsen, Anders Elm Pedersen, Karen Angeliki Krogfelt, Andreas Munk Petersen

**Affiliations:** 1 Department of Gastroenterology, Copenhagen University Hospital Hvidovre, Copenhagen, Denmark; 2 Department of Obstetrics and Gynecology, Copenhagen University Hospital, Hvidovre, Copenhagen Denmark; 3 Faculty of Health and Medical Sciences, University of Copenhagen, Copenhagen, Denmark; 4 Department of Pediatrics, Copenhagen University Hospital Hvidovre, Copenhagen, Denmark; 5 Department of Epidemiology Research, Statens Serum Institut, Copenhagen, Denmark; 6 Faculty of Food Science and Nutrition, University of Iceland, Reykjavik, Iceland; 7 Department of Immunology and Microbiology, Faculty of Health and Medical Sciences, University of Copenhagen, Copenhagen, Denmark; 8 Department of Microbiology and Infection Control, Statens Serum Institut, Copenhagen, Denmark; 9 Department of Clinical Microbiology, Copenhagen University Hospital Hvidovre, Copenhagen, Denmark

**Keywords:** Study protocol, Pregnancy, Obesity, Microbiota, Probiotics, Vivomixx®, Gestational diabetes mellitus

## Abstract

**Background:**

Maternal obesity is associated with increased risks of adverse pregnancy-related complications and outcomes for both mothers and infants. Overweight and obese women have an increased risk of pregnancy-induced hypertension, preeclampsia and gestational diabetes mellitus (GDM). Infant Body Mass index (BMI) and the risk of obesity in adulthood are related to maternal gestational weight gain (GWG). Preventive lifestyle and dietary interventions are time-consuming and do not always reduce GWG or the risk of maternal pregnancy complications. Recent research has indicated that the gut microbiota may play a significant role in the development of obesity. Some studies have indicated that the daily consumption of probiotics may reduce the risk of preeclampsia, maintain serum insulin levels and reduce the frequency of GDM in pregnant women. The aims of this study are to investigate whether daily probiotic supplements in obese women during pregnancy can limit gestational weight gain, improve glucose homeostasis and thereby improve maternal, fetal and infant health outcomes.

**Methods:**

A pilot study including 50 obese pregnant nulliparous women with a prepregnancy BMI of between 30 and 35 kg/m^2^ will be randomized to receive daily probiotics (four capsules of Vivomixx®; total of 450 billion CFU/day, including eight probiotic bacterial strains) or placebo from gestational age 14–20 weeks until delivery. The infants will be followed until 9 months of age. The women will be monitored by weight, blood, fecal, vaginal and urine samples, diet questionnaires and hospital record review. Primary outcomes are: maternal weight gain, glycated hemoglobin (HbA1c) level and changes in glucose concentration measured during an oral glucose tolerance test. Secondary outcomes are: microbiota and inflammatory markers in mother and child, pregnancy complications, pregnancy outcomes, physical activity and the body composition of the neonate.

**Discussion:**

We expect to find alterations in the metabolic profiles, microbiota and possibly pregnancy outcomes. From a clinical point of view the effects of Vivomixx® could control weight gain and reduce complications during pregnancy by inducing changes in the gut microbiota. Furthermore, this intervention during pregnancy could influence the infant’s microbiota, which could have important implications for infant development and health.

**Trial registration:**

ClincalTrials.gov Identifier: NCT02508844, registered on 11 May 2015.

**Electronic supplementary material:**

The online version of this article (doi:10.1186/s13063-016-1617-5) contains supplementary material, which is available to authorized users.

## Background

Maternal obesity has become highly prevalent worldwide and is associated with an increased risk of maternal pregnancy-related complications and an increased risk of adverse pregnancy outcomes [1]. Women who are overweight or obese during pregnancy and childbirth, as measured by a high maternal Body Mass Index (BMI), have an increased risk of pregnancy-induced hypertension, preeclampsia and gestational diabetes mellitus (GDM) [2]. Maternal obesity may adversely affect the fetus and fetal growth in the initial stages of life, the risk of pregnancy complications, as well as the child’s later development [3]. Maternal GDM increases the risk of excessive adiposity in the fetus, macrosomia (a birth weight of over 4000 g) and neonatal hypoglycemia [4, 5]. In the long term, maternal GDM is associated with an increased risk of obesity as well as metabolic (type 2 diabetes) and cardiovascular disease in both mother and child [6]. Over the past three decades, the incidence of obesity among children and adolescents has increased radically [7]. Gestational weight gain (GWG) is related to BMI in childhood and to the risk of obesity in adulthood [8]. The newborn’s fat mass increases by every kilogram of GWG [9], but in obese women adherence to GWG recommendations do not prevent high birth weights [10]. In obese pregnant women, a randomized study has shown that physical activity intervention assessed by pedometer reduces GWG compared with controls, but also that the recommended GWG of maximal 9 kg was only met in about 50 % of the women [11].

Most studies addressing the prevention of GDM and excessive GWG among overweight and obese pregnant women have been restricted to lifestyle interventions, which often result in either no or modest reduction in GWG [12].

Probiotics are live microorganisms which, when administered in adequate amounts, may confer a health benefit on the host [13]. Probiotics consist of individual or multiple live bacterial species (such as lactobacilli and bifidobacteria) that during intake can alter the gut microbiota [14]. Recent research has indicated that the gut microbiota may play a significant role in the development of obesity, obesity-associated inflammation and insulin resistance [15]. Experiments with mice have shown that the composition of the bacteria in the gut may affect weight regulation [16]. In other animal models, associations have been found between changes in gut flora and obesity, insulin resistance and diabetes [17]. Modulation of the gut microbiota early in life has thus attracted interest since differences in its compositional development may predict the risk of overweight in the offspring [18].

The etiology of the development of obesity is not clearly elucidated, but a complex interaction between genetic, epigenetic and social factors is likely. The prevalence of obesity has been rising faster than can be explained by biological variation, indicating that genetics is not the only cause [19]. In addition, there is small but consistent evidence showing that the daily consumption of probiotics may reduce the risk of preeclampsia [20], maintain serum insulin levels [21] and reduce the frequency of GDM [22] in pregnant women. Vivomixx® consists of eight strains of freeze-dried probiotic bacteria. The product contains the same mixture of bacteria as the product VSL#3 and some studies demonstrate that VSL#3 may normalize gut permeability and barrier function, which is associated with beneficial anti-inflammatory and immunomodulatory properties [23, 24]. A new study on mice has demonstrated that the administration of VSL#3 can prevent and treat obesity and diabetes in several mouse models and suppress body weight gain and insulin resistance via modulation of the gut microbiota [25]. VSL#3 promotes release of the hormone glucagon-like peptide 1 (GLP-1) resulting in reduced food intake and improved glucose tolerance. The VSL#3-induced changes are associated with an increase in the levels of a short chain fatty acid (SCFA), butyrate [24]. Butyrate stimulates the release of GLP-1 from intestinal L-cells thereby providing a plausible mechanism for VSL#3’s action. Promising results have also been shown in humans. A randomized controlled study by Rajkumar et al. in 2014 among 60 overweight (BMI >25) but otherwise healthy adults showed a significant reduction in total cholesterol, triglycerides, low density lipoproteins (LDL) and very-low-density lipoproteins (VLDL) (*P* < 0.05) after 6 weeks of VSL#3 treatment. They also found improved insulin sensitivity (*P* < 0.01), decreased C-reactive protein (CRP), and a favorable effect on the composition of gut microbiota [26]. A randomized study by Alisi et al. in 2014 examined the beneficial effects of VSL#3 in 48 obese children with nonalcoholic steatohepatitis. After 4 months of treatment, BMI was significantly reduced and GLP-1 secretion increased in the VSL#3-supplemented children compared to the placebo group (*P* < 0.001) [27]. Moreover, VSL#3 has also been tested in a small interventional study among 27 healthy pregnant women by Vitali et al. in 2012 in order to investigate changes in the composition of the vaginal microbiota and cytokine secretion. VSL#3 was administered during the last trimester of pregnancy and was associated with modulation of the vaginal microbiota and cytokine secretion [28]. None of the studies [25–27] reported side effects related to the treatment with VSL#3. As accumulating evidence indicates that the gut microbiota plays a significant role in obesity and because the “ideal” composition of the gut microbiota remains poorly understood, modulation of the gut microbiota composition represents a potentially attractive treatment option against excessive GWG and adverse outcomes for mothers and infant. Dietary supplements in the form of probiotics could potentially be beneficial for controlling weight gain in pregnancy by inducing changes in the gut microbiota and could also affect the infant’s microbiota, which may have important implications for infant development and health.

### Aims and hypotheses

In a pilot study of 50 obese pregnant women, we aim to investigate if the probiotic Vivomixx® can affect gestational weight gain, glycated hemoglobin (HbA1c) level and reduce impairment of glucose tolerance at gestational weeks 27–30, compared to weeks 14–20, in obese pregnant women. The aim of conducting an initial pilot study (*n* = 50) is to clarify feasibility and compliance and to estimate parameters, such as the standard deviation, which will be used in a sample size calculation for a full-scale trial. The study will also give an estimate of the proportion of eligible women who are willing to participate, of participants who drop out of the trial and of participants who comply with their allocated intervention.

Secondary aims are:

- To examine changes in microbiota and inflammatory markers in mother and child (in fecal samples)
- To examine changes in vaginal microbiological profile and frequency of urinary tract infections (including group B streptococcus infections)
- To examine changes in concentrations of lipids and inflammatory markers (maternal blood sample)
- To assess if the intervention reduces the risk of complications during pregnancy and at birth – including GDM, preeclampsia, gestational hypertension, change in the mode of delivery, gestational age, macrosomia (birth weight of over 4000 g) and large- and small-for-gestational-age infants
- To examine the impact of the intervention on diet, physical activity levels and breastfeeding
- To examine differences in the neonatal outcome – weight, z-score, Apgar score after 5 min, umbilical cord pH, and the risk of early neonatal transfer to the neonatal intensive care unit
- To assess the child’s weight gain and body composition until 9 months of age (dual-energy X-ray absorptiometry (DEXA) scanning (at birth) and skin-fold measurements thereafter)

## Methods

### Study design

**Fig. 1:**
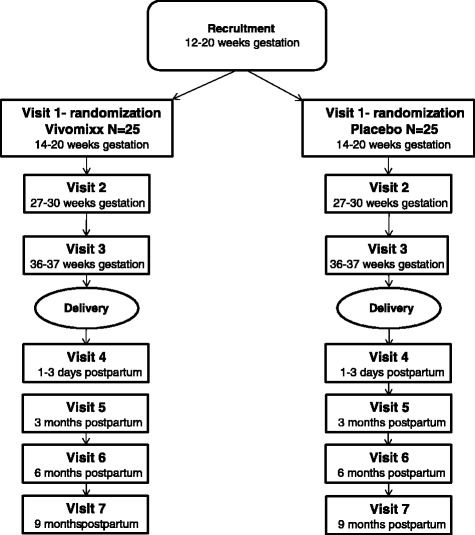
Study flow chart of the pilot study

In this single-center, double-blind, randomized, placebo-controlled pilot study, 25 obese pregnant women will be treated with probiotics and 25 will be treated with placebo. Included pregnant women will receive two capsules of the probiotic mixture Vivomixx® or placebo twice daily from gestational weeks 14–20 until delivery. Examination including blood, urine, vaginal and fecal sampling will be performed at gestational weeks 14–20 (baseline), 27–30 and 36–37 in all study participants. Data on infants are collected at birth, 18–72 h after birth, and after 3, 6 and 9 months. An overview of study visits is illustrated in Fig. 1. The study will connect to the study “The Treatment of Obese Pregnant Women (TOP)” [11] at Hvidovre University Hospital, with the objective to compare data.

### Recruitment of participants

The women will be identified when they are booked for the initial nuchal translucency ultrasound scan at Copenhagen University Hospital Hvidovre. Written consent will also be obtained from obese pregnant women who do not wish to participate in order to compare data from their pregnancy outcome with that of the study participants.

### Inclusion criteria

- Aged over 18 years
- Prepregnancy BMI ≥30 and <35 kg/m^2^
- Nulliparous singleton pregnancy
- Ability to read and speak Danish
- Normal ultrasound scan of the fetus at gestational age 12–14 weeks
- Oral glucose tolerance test (OGTT) at gestational age 14–20 weeks

### Exclusion criteria

- Pregnancy at over 20 weeks’ gestation at recruitment
- Pregestational diabetes or other serious diseases
- Multiple pregnancy
- Previous bariatric surgery
- Ingestion of probiotics more than 1 month before the inclusion or ingestion of probiotics other than the study probiotics
- Alcohol or drug abuse

### Randomization procedure

The women are randomized to Vivomixx® or placebo in a 1:1 ratio. The capsules are packed in numbered bags with either Vivomixx® or placebo content at the hospital pharmacy (Region Hovedstadens Apotek, Klinisk Farmaceutisk Service, Kettegaard Alle 30, 2650 Hvidovre). The randomization is done by computer software in blocks of 4. Both capsules are identical in appearance and packages. The identity of the capsules is unknown to participants, researchers and primary investigators.

### Study agent

Vivomixx® (manufactured by Mendes SA, Lugano, Switzerland) is a probiotic mixture which contains the following strains: *Streptococcus thermophilus* DSM 24731, bifidobacteria (*Bifidobacterium breve* DSM 24732, *Bifidobacterium longum* DSM 24736, *Bifidobacterium infantis* DSM 24737) and lactobacilli (*Lactobacillus acidophilus* DSM 24735, *Lactobacillus plantarum* DSM 24730, *Lactobacillus paracasei* DSM 24733, *Lactobacillus delbrueckii* subsp. *bulgaricus* DSM 24734) and is formulated in vegetable capsules containing 112 billion lyophilized bacteria. Women allocated to the placebo group will receive capsules containing microcrystalline cellulose, magnesium stearate and silicon dioxide. Capsules are stored at 2 to 8 °C prior to distribution. The participants are also instructed to refrigerate the capsules at home. Both written and verbal instructions regarding use and storage of capsules are provided.

### Data handling and record keeping

Case Report Forms (CRF) will be used to record data for all participants, and data will be double-entered on an electronic database to secure its validity. Questions in the CRF collect a range of data including effect modifiers (antibiotics), outcome data and adverse events. The research staff is the same two people throughout the study to standardize the data collection procedure.

### Assessment of primary outcomes

Gestational weight gain is the primary outcome. Maternal weight gain is defined as weight at gestational age 36–37 weeks minus self-reported prepregnancy weight. All women are weighed at the same scale at every study visit, wearing light clothing and no shoes (Seca digital scales, Seca, Hamburg, Germany). GWG values are calculated as the difference between the mother’s prepregnancy weight and her weight at 36–37 weeks of gestation.

The other primary outcome is the change in maternal fasting glucose from weeks 14–20 (preintervention) to weeks 27–30 between probiotic and placebo groups. GDM outcomes are assessed at 14–20 and 27–30 weeks’ gestation by a three-time-point 75-g OGTT. Preparation for the test includes a 10–13-h pretest fast with instructions to avoid smoking and chewing gum during this time and during the test. Tap water intake should be limited to two glasses (300 ml) and exercise on the morning of the test should be avoided. Participants remain in a resting state during the test.

Measurements are listed in Table 1.

**Table 1:** Maternal and neonatal/infant primary and secondary outcomes

Measurement	Inclusion/randomization (14–20 weeks)	27–30 weeks	36–37 weeks	Delivery	1–3 days after delivery	3 months after delivery	6 months after delivery	9 months after delivery
Baseline questionnaire	X							
Diet questionnaire	X		X					
Weight (pregnant/mother)	X	X	X		X	X	X	X
Blood sample (pregnant/mother)	X	X	X		X	X		X
Blood pressure	X	X	X					
Fecal sample (pregnant/mother)	X	X	X		X	X	X	X
Urine sample + vaginal swab	X	X	X					
Pedometer	X	X	X					
OGTT	X	X						
Fecal sample – infant					X	X	X	X
Urine sample – infant					X	X	X	X
Newborn – umbilical cord blood sample				X				
DEXA scanning					X			
Breast milk sample					X	X	X	X
Newborn/child physical examination and anthropometric data					X	X	X	X
Question about urinary tract infections	X	X	X					
Question about colic symptoms, breastfeeding, allergy and atopic dermatitis						X	X	X

*DEXA* dual-energy X-ray absorptiometry, *OGTT* oral glucose tolerance test

### Other measures

#### Questionnaires

Participants in the study will complete a questionnaire at the time of inclusion about demographic data including smoking history, educational level, and data about prepregnancy physical activity. Women who decline to participate in the study will be asked to complete the same questionnaire. Dietary and supplemental intake will be measured by a validated Food Frequency Questionnaire (FFQ) at the time of inclusion and before delivery (weeks 36–37) [29].

#### Physical activity

Participants will be instructed in using a validated pedometer: a Yamax Digiwalker CW-700/750 (Yamax Corporation, Tokyo, Japan) [30] three times during pregnancy to monitor physical activity. Daily step counts will be registered on seven consecutive days at inclusion (baseline) and in weeks 27–30 and weeks 36–37 of gestation, and the women will also be asked about swimming and cycling during pregnancy.

#### Fecal samples

Fecal samples are collected at home and sent by mail and then frozen at −80 °C. Bacterial deoxyribonucleic acid (DNA) will be extracted from the fecal samples using the PowerSoil DNA Isolation Kit, which has been found to be suitable in similar clinical studies [31]. The universal bacterial primers S-D-Bact-0341-b-S-17 and S-D-Bact-0785-a-A-21, affixed to Illumina adapters, will be used for the amplification of the 16S V3-V4 region. This primer couple has been found to be appropriate to monitor microbe diversity [32].

#### Urine samples

Urine samples are collected at home by the participant on the morning that they attend the study visit. The sample should be taken from first midstream urine in the morning after overnight fasting. At the hospital 5 ml are frozen at −80 °C in sterile tubes (Falcon 15-ml polypropylene) containing 50 μl NaN_3_.

#### Vaginal samples

Vaginal samples are collected at home using an eSwab and sent by mail. eSwab is a liquid-based multipurpose collection and transport system that maintains the viability of aerobic, anaerobic and fastidious bacteria for up to 48 h.

#### Blood samples

Blood samples will be taken at every study visit during the study period. They will be analyzed for concentrations of i.a. lipids and lipoproteins (high-density lipoprotein (HDL), low-density lipoprotein (LDL) and total cholesterol), inflammatory markers (CRP, interleukin (IL)-18, IL-6 and tumor necrosis factor alpha (TNF-α)), Glycated hemoglobin (HbA1c) level, homocysteine, GLP-1 and BDNF (brain-derived neurotrophic factor). At study visits during pregnancy the participants will be fasting.

#### Breast milk samples

Breast milk samples will be collected at all study visits after birth. Samples are taken on the day of the study visit. The samples are frozen at −80 °C at the hospital.

### Hospital chart review

Hospital records directly related to pregnancy and delivery will be reviewed. Pregnancy complications and mode of delivery will be extracted from hospital files and validated using the Danish national guidelines. GDM will be defined according to the International Association of Diabetes in Pregnancy Study Group (IADPSG) criteria, to be able to compare results from this study with other international studies. Preeclampsia will be defined as proteinuria (dipstick, greater than 1+ protein) and persistently elevated blood pressure greater than 140/90 mmHg on more than one occasion. Gestational hypertension is diagnosed using the same criteria but without proteinuria. Macrosomia is defined as birthweight of 4000 g or greater. The z-score of the newborn will be calculated (deviation from the expected weight corrected for gestational age and sex) [33]. Apgar scores and eventual delivery complications will also be registered. All admissions to the neonatal care unit will also be recorded along with diagnosis for admission. Women who choose to withdraw from the study will be asked if they will allow review of their hospital records for the purpose of comparing “drop-outs” with women who remain in the study.

### DEXA scanning and infant examination

A DEXA scan is conducted before 72 h post birth for the newborn and the parents. The method is validated in the newborn [34]. The infants will be weighed recumbent (Seca 727, digital baby scales, Seca, Hamburg, Germany), and their length and head circumference are measured with a measuring tape according to World Health Organization guidelines. Abdominal circumference is measured at the umbilical level in the supine position during mid-expiration. Newborn anthropometric measurements are collected from their birth chart.

### Subject compliance monitoring and capsule viability

Patient compliance will be monitored by requesting the patients to return all unused capsules at the visit at gestational weeks 27–30 and at follow-up after delivery. The patient should consume at least 80 % of the study medication during the study period. Fecal samples are collected from participants and it will be proven whether the probiotic bacteria are established in the gut of the intervention group by using strain-specific primers. Samples of capsules returned from the participants will be tested to ensure that the capsules have maintained their viability.

### Safety and adverse event monitoring

Probiotic bacteria are widely occurring intestinal bacteria and interventional studies with probiotic bacteria are generally considered to be safe. Probiotic bacteria are used in fermented dairy products and other foods including some infant formulas. Moreover, there is a wide range of commercial products available at pharmacies and health food stores.

All strains included in Vivomixx® are known and accepted organisms in food and have been used in clinical studies in the form of VSL#3 in obese children with nonalcoholic steatohepatitis [27], patients with inflammatory bowel disease [35–37], critically ill patients [38], patients with cirrhosis [39, 40] and pediatric patients [41, 42] with no reported side effects.

Clinical studies on pregnant women and infants also support the safe use of probiotics during pregnancy and early infancy [22, 43, 44].

Participants are asked about side effects of the product at every study visit and their answers are recorded in the CRF.

### Sample size and statistics

This is the first pilot study in which the probiotic Vivomixx® is added in a randomized fashion to the standard of care in obese pregnant women. Fifty pregnant women are planned to be included. Data for publication of these data will be analyzed with statistical software and significance will be set at *P* < 0.05.

By a group size of 2 × 25 participants, and on the assumption that probiotics reduce average GWG by 2 kg – from 10 kg to 8 kg (SD 6.5), the study will have a strength of 19 %. Under these assumptions the expected width of the confidence interval will be 5.

All continuous data results based on treatment with Vivomixx® and placebo will be analyzed using chi-square, and all categorical data results based on treatment with Vivomixx® or placebo will be analyzed using *t* tests.

For planning of the final study the significance level for the outcomes will be set at *P* < 0.1 to select the relevant outcome measurements.

## Discussion

In summary, previous studies in pregnant obese women state that obesity in pregnancy is associated with adverse outcomes for both mothers and infants, including the risk for pregnancy-induced hypertension, preeclampsia, GDM and macrosomia. Weight gained in pregnancy affects the health of both the mother and her unborn child. Most studies for the prevention of GDM and excessive GWG among overweight and obese pregnant women have until now been restricted to lifestyle interventions and have shown mixed results and only modest improvements [12]. Probiotic supplementation, if beneficial, would be easier to use in clinical practice. As previously mentioned, evidence exists that daily consumption of probiotics could reduce the risk of preeclampsia [20], maintain serum insulin levels [21] and reduce the frequency of GDM [22] in pregnant women. These trials used different probiotic strains, and it is a general assumption that different kinds of probiotics influence the microbiota differently and thereby have varying effects on metabolic functions. The study by Luoto et al. in 2010 was a randomized controlled trial of probiotic intervention with capsules containing *Lactobacillus rhamnosus* GG and *Bifidobacterium lactis* BB12 in 256 normal-weight pregnant women and reported a reduced risk of GDM from 34 to 13 % (*P* = 0.003) with a combined dietary/probiotic supplementation [22]. Two other studies have examined the effect of probiotic milk products. The study by Asemi et al. in 2013 was a randomized controlled trial including 70 pregnant women in their third trimester who were randomly assigned to consume 200 g per day of conventional or probiotic yoghurt containing two strains of lactobacilli (*L. acidophilus* LA5) and bifidobacteria (*Bifidobacterium animalis* BB12) for 9 weeks. Significant differences were found in serum insulin levels: +1.2 ± 1.2 versus +5.0 ± 1.1 μIU/ml (*P* = 0.02), and the conclusion was that the consumption of probiotic yoghurt maintains serum insulin levels and might help prevent developing insulin resistance during pregnancy [21]. Brantsæter et al. in 2011 studied a cohort of 33,399 primiparous women where milk-based products containing probiotic lactobacilli (*L. acidophilus* LA5, *B. lactis* BB12, *L. rhamnosus* GG) were estimated from a self-reported FFQ. Adjusted for confounders, intake of probiotic products during pregnancy was associated only with reduced risk of severe preeclampsia (odds ratio = 0.79, 95 % confidence interval 0.66, 0.96) [20]. Only one double-blind, placebo-controlled, randomized study, using probiotics in 175 pregnant obese women has so far been carried out but showed no effects on either maternal fasting glucose, metabolic profile or pregnancy outcomes after 4-week probiotic treatment (*Lactobacillus salivarius* UCC118) between gestational weeks 24 and 28 [45]. The duration of the intervention could influence the outcomes of the studies, and 4 weeks is possibly too short a period to induce significant changes as infants receive their first microbial flora at the time of delivery. These inoculated bacteria reflect the microbiota of the mother’s vagina and gastrointestinal tract and influence further intestinal microbiota development [46]. The gut microbiotas of infants are dynamic and highly dependent on a number of factors including delivery method and diet (for example, breastfeeding versus formula-feeding) [47]. Differences in the initial microbiota, e.g. gut microbiota associated with overweight or excessive weight gain, can alter the developmental pathways of the infant’s microbiota, which could have important implications for infant development and health [48]. A recently published systematic review of studies that reported on the effects on maternal outcomes of probiotic treatment in pregnant women highlighted the need for studies involving obese women because of their increased risk of adverse pregnancy outcomes [49]. Also a review by Barrett et al. in 2014 [50] on probiotics for preventing GDM concluded that there is a requirement for further studies – especially in populations with higher risks of developing GDM. Because maternal obesity is highly prevalent worldwide and associated with adverse outcomes for both mothers and infants, it is important to develop new treatment methods to prevent these adverse outcomes. Intrauterine life and early infancy are critical stages for targeting interventions aiming to reduce the risk of overweight development in future generations. We hypothesize that changing the microbial environment of the mother and her fetus can modify the health of the child. The findings of this study should advance current knowledge in this field in terms of weight management interventions for obese pregnant women, as well as the influence of probiotics on pregnancy and on the child’s health. In summary, we expect that our findings will demonstrate that Vivomixx® can alter gut microbiota in obese pregnant women and thereby reduce the risk of developing pregnancy and birth complications. We anticipate that results from this trial may have implications for the future treatment of obese pregnant women. The intervention could result in fewer obese children, with significant importance for the individual child healthwise, psychologically and socially. The results will also form the basis for future research to ensure healthy mothers and children.

Results from this pilot study will provide data to facilitate planning for a definitive study investigating the effects of Vivomixx® in pregnant obese women and their newborn.

## Trial status

This pilot study started enrolling in March 2015. Currently, 20 participants have been included in the study (November 2015).

## Additional file

Additional file 1: SPIRIT check list. (DOC 121 kb)

## Abbreviations

BMI: Body Mass index
CRF: Case Report Form
CRP: C-reactive protein
FFQ: Food Frequency Questionnaire
GDM: Gestational diabetes mellitus
GWG: Gestational weight gain
HbA1c: Glycated hemoglobin
HDL: high-density lipoprotein
IADPSG: International Association of Diabetes in Pregnancy Study Group
LDL: Low-density lipoprotein
OGTT: Oral glucose tolerance test
VLDL: Very-low-density lipoprotein
